# Multimodal analyses of vitiligo skin idenitfy tissue characteristics of stable disease

**DOI:** 10.1172/jci.insight.154585

**Published:** 2022-07-08

**Authors:** Jessica Shiu, Lihua Zhang, Griffin Lentsch, Jessica L. Flesher, Suoqin Jin, Christopher Polleys, Seong Jin Jo, Craig Mizzoni, Pezhman Mobasher, Jasmine Kwan, Francisca Rius-Diaz, Bruce J. Tromberg, Irene Georgakoudi, Qing Nie, Mihaela Balu, Anand K. Ganesan

**Affiliations:** 1 Department of Dermatology,; 2 Department of Mathematics,; 3 NSF-Simons Center for Multiscale Cell Fate Research, and; 4 Beckman Laser Institute and Medical Clinic, University of California, Irvine (UC Irvine), Irvine, California, USA.; 5 Cutaneous Biology Research Center, Department of Dermatology, Massachusetts General Hospital, Boston, Massachusetts, USA.; 6 Department of Biomedical Engineering, Tufts University, Medford, Massachusetts, USA.; 7 Department of Dermatology, Seoul National University College of Medicine, Seoul, Republic of Korea.; 8 Department of Preventive Medicine and Public Health, University of Malaga, Malaga, Spain.; 9 Department of Developmental and Cell Biology and; 10 Skin Biology Resource Center, UC Irvine, Irvine, California, USA.

**Keywords:** Autoimmunity, Dermatology, Diagnostic imaging, Expression profiling, Skin

## Abstract

Vitiligo is an autoimmune skin disease characterized by the destruction of melanocytes by autoreactive CD8^+^ T cells. Melanocyte destruction in active vitiligo is mediated by CD8^+^ T cells, but the persistence of white patches in stable disease is poorly understood. The interaction between immune cells, melanocytes, and keratinocytes in situ in human skin has been difficult to study due to the lack of proper tools. We combine noninvasive multiphoton microscopy (MPM) imaging and single-cell RNA-Seq (scRNA-Seq) to identify subpopulations of keratinocytes in stable vitiligo patients. We show that, compared with nonlesional skin, some keratinocyte subpopulations are enriched in lesional vitiligo skin and shift their energy utilization toward oxidative phosphorylation. Systematic investigation of cell-to-cell communication networks show that this small population of keratinocyte secrete CXCL9 and CXCL10 to potentially drive vitiligo persistence. Pseudotemporal dynamics analyses predict an alternative differentiation trajectory that generates this new population of keratinocytes in vitiligo skin. Further MPM imaging of patients undergoing punch grafting treatment showed that keratinocytes favoring oxidative phosphorylation persist in nonresponders but normalize in responders. In summary, we couple advanced imaging with transcriptomics and bioinformatics to discover cell-to-cell communication networks and keratinocyte cell states that can perpetuate inflammation and prevent repigmentation.

## Graphical abstract

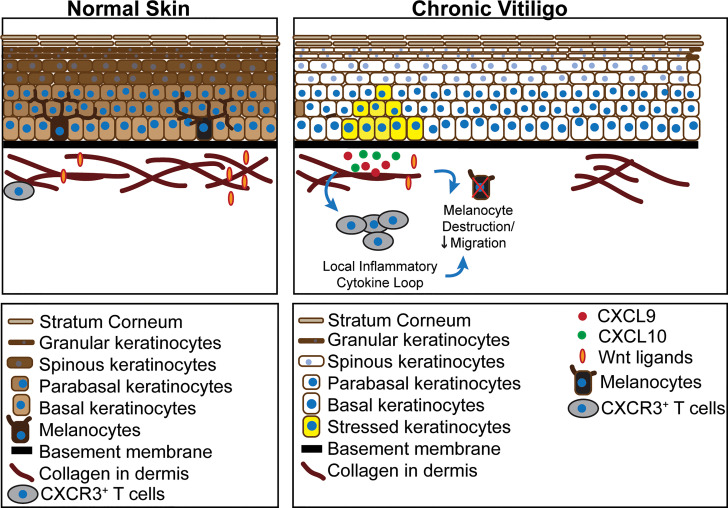

## Introduction

Vitiligo is an autoimmune skin disease characterized by the progressive destruction of melanocytes by autoreactive CD8^+^ T cells, resulting in disfiguring patches of white depigmented skin that cause significant psychological distress among patients (1). CD8^+^ T cells play an important role in the elimination of melanocytes and are increased in active vitiligo skin (2–4). However, in stable vitiligo lesions devoid of melanocytes, T cells are sparse and immune activation levels are low (5). This makes it unclear why white patches continue to persist in the absence of a robust inflammatory infiltrate.

Development of mouse models representative of human disease has provided important clues regarding the role of the adaptive immune system in vitiligo (6, 7). Keratinocytes secrete CXCL9 and CXCL10 to attract and activate CXCR3^+^CD8^+^ T cells (8), and these chemokines are present in the blister fluid of human vitiligo patients (4). However, the adoptive transfer of autoreactive CD8^+^ T cells in the mouse model cannot fully recapitulate the complex interactions between melanocytes, keratinocytes, and immune cells that occurs in situ in human skin. Melanocytes are present in the epidermis in only select locations in mice (9), and the mouse epidermis is considerably thinner and lacks the stratification seen in human skin (10). To date, most translational studies in vitiligo are limited to examining cultured cells in vitro or IHC of diseased tissue (11). It has been difficult to study how cell lineages collectively contribute to disease persistence secondary to the lack of tools to assess cellular heterogeneity in vivo.

Multiphoton microscopy (MPM) is a unique tool for this purpose and has broad applications in human skin (12–19). MPM is a noninvasive imaging technique capable of providing images with submicron resolution and label-free molecular contrast, which can be used to characterize keratinocyte metabolism in human skin (20, 21). This approach is based on the 2-photon excited fluorescence (TPEF) signal detected from the reduced nicotinamide adenine dinucleotide (NADH), a coenzyme in the keratinocyte cytoplasm that plays a central role in metabolism. We have validated this technique’s ability to assess cellular metabolism in normal skin under hypoxic conditions (21, 22). Specifically, we have shown that the intensity fluctuations from NADH TPEF images can be analyzed to reveal changes in mitochondrial organization and dynamics in a highly sensitive manner (21–23). This is possible because the NADH fluorescence yield is enhanced 10-fold when NADH is bound in the mitochondria, instead of in its free form in the cytosol (24). Since the organization of mitochondria in a fragmented or networked state is highly sensitive to metabolic function (25), the level of mitochondrial clustering (or fragmentation) that we derive from analysis of NADH TPEF images can serve as a quantitative metric of metabolic function. Indeed, we have detected significant changes in mitochondrial clustering in response to changes in the relative levels of several important metabolic pathways, including glycolysis, oxidative phosphorylation (OxPhos), and fatty acid oxidation and synthesis (23). We have further demonstrated that this type of analysis is sensitive to changes in the relative levels of OxPhos and glycolysis that are present along the depth of normally differentiating squamous epithelial tissues, such as that of the skin and the cervix (21, 26). Importantly, we have validated this approach by detecting dynamic changes in mitochondrial clustering of human skin epithelia confined to the basal layer in response to hypoxia, consistent with an expected enhancement in the relative levels of glycolysis (21).

In this study, we employ MPM for in vivo imaging of stable vitiligo lesions and assess the keratinocyte metabolic state based on an imaging metric derived from a mitochondrial clustering analysis approach validated in previous studies (21, 22). We then performed single-cell RNA-Seq (scRNA-Seq) on patient-matched lesional and nonlesional tissue to identify keratinocyte subpopulations in stable vitiligo and apply CellChat to analyze intercellular communication networks in scRNA-Seq data. We demonstrate that stress keratinocytes communicate with adaptive immune cells via the CXCL9/CXCL10/CXCR3 axis to create local inflammatory loops that are active in stable vitiligo. Moreover, signaling between melanocyte and keratinocytes via the WNT pathway was altered in stable vitiligo lesions. We implicate a role for stress keratinocytes in disease persistence by showing that they normalize their metabolic signals and resemble nonlesional skin keratinocytes in patient skin that responds to punch grafting treatment. By integrating noninvasive MPM, scRNA-Seq, and advanced bioinformatics, we infer communication networks between keratinocytes, melanocytes, and immune cells capable of preventing normal melanocyte repopulation.

## Results

### MPM imaging of stable vitiligo skin in vivo demonstrates mitochondrial clustering changes.

**Figure 1:**
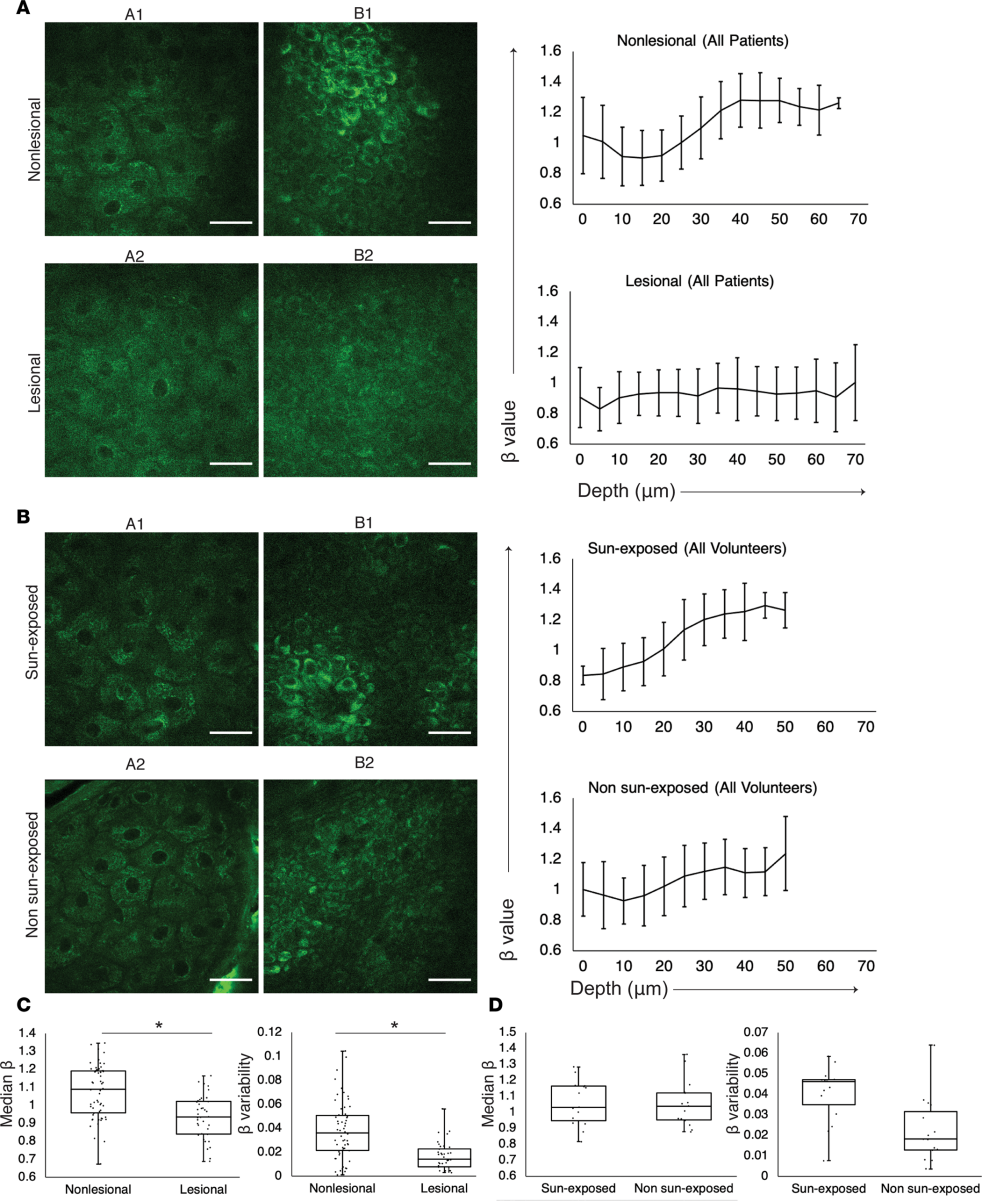
In vivo MPM images of vitiligo lesional and nonlesional skin showing metabolic changes with depth independent of sun exposure. (**A**) Representative en-face MPM images from the stratum granulosum in nonlesional (A1) and lesional skin (A2) and from the basal layer in nonlesional (B1) and lesional skin (B2) of 1 vitiligo patient. Average mitochondrial clustering values (β values) based on *Z* stacks from all vitiligo patients (*n* = 12) as a function of depth for nonlesional (top right) and lesional (bottom right) skin are shown as spline fits. Data are shown as mean ± SD of the measurements for the images in all the *Z* stacks at each area. The labels A1, A2, B1, and B2 within the mitochondrial clustering panels represent the mitochondrial clustering values extracted from the panel’s respective labeled images. Scale bars: 20 μm. (**B**) Representative en-face MPM images from the stratum granulosum in sun-exposed (A1) and non–sun-exposed skin (A2) and from the basal layer in sun-exposed (B1) and non–sun-exposed skin (B2) of 5 healthy volunteers. (**C**) Distribution of the median β values (left) and β variability values (right) in nonlesional and lesional skin of 12 vitiligo patients; each value corresponds to a *Z* stack of images acquired in nonlesional and lesional skin. *P < 0.05 by 2-tailed *t* test.(**D**) Distribution of the median β values (left) and β variability values (right) in sun-exposed and non–sun-exposed skin of 5 healthy volunteers; each value corresponds to a *Z* stack of images acquired in non–sun-exposed and sun-exposed areas.

To look at epidermal changes using MPM in stable vitiligo, we utilized the MPTflex clinical microscope to image 12 patients with lesions characterized by depigmented areas that have not grown in size for at least 1 year and did not exhibit active vitiligo features such as confetti-like depigmentation, koebnerization, and trichome (Supplemental Table 1; supplemental material available online with this article; https://doi.org/10.1172/jci.insight.154585DS1) (27). As expected, MPM images of nonlesional skin showed brighter fluorescence spots in the cellular cytoplasm, which represent aggregates of melanosomes, compared with lesional skin (Figure 1A) (15). To evaluate for metabolic changes in nonlesional and lesional vitiligo skin, we studied mitochondrial clustering that was previously validated in skin under normal and hypoxic conditions (21). Consistent with published data, nonlesional skin exhibited depth-dependent changes in mitochondrial clustering that reflects differences in metabolism (Figure 1A). In short, the basal and parabasal keratinocytes present a fragmented mitochondria phenotype characterized by high values of the mitochondrial clustering metric, β. As cell differentiation progresses from the basal to the higher epidermal layers and cells turn from glycolysis to OxPhos for energy production, mitochondria fuse and create more extensive networks that correspond to lower clustering values, reaching their minima within the spinous layer (Figure 1A). Finally, toward the most terminally differentiated layer, as the granular keratinocytes enter an apoptotic state to create the stratum corneum, mitochondrial clustering values recover again, signifying a return to a more fissioned phenotype. In contrast, lesional depigmented skin from vitiligo patients showed an altered trend of mitochondrial clustering compared with nonlesional skin (Figure 1A), suggesting that the depth-dependent metabolic changes were lost. We calculated the mitochondrial clustering (β) median value and its variability across the epidermis of vitiligo and normal skin, and we found that these metrics are significantly different in vitiligo lesional and nonlesional skin (Figure 1C). Given that these changes were observed in the basal layer, we performed an additional analysis to compare mitochondrial clustering between lesional and nonlesional basal keratinocytes. This analysis indicates a more heterogeneous distribution of mitochondrial clustering value (β values) for lesional vitiligo versus nonlesional basal keratinocytes (Supplemental Figure 1), yielding distributions with heterogeneity index values of 0.16 and 0.12, respectively. Noticeably, vitiligo basal keratinocytes exhibited an increase in the number of cells characterized by lower mitochondrial fragmentation levels and, thus, more networked mitochondria, consistent with enhanced OxPhos (21–23).

Since the fluorescence signals from all the skin fluorophores, including NADH, are collected on the same detection channel in the MPTflex, we sought to ensure that the mitochondrial clustering measurements were not affected by contributions from fluorophores other than NADH. Melanin requires particular consideration, since it is the main source of difference in appearance between vitiligo and normal skin. To ensure that melanin content was effectively removed and not affecting fluorescence signal analysis sensitivity to mitochondrial dynamics, we measured mitochondrial clustering in 5 healthy volunteers. We controlled for melanin content by comparing sun-exposed sites (dorsal forearm) and non–sun-exposed sites (volar upper arm, which would have relatively less melanin). We found that depth-dependent β values showed similar trends in the epidermis (Figure 1B), regardless of sun-exposure status, and that the median β values and β variability values were not significantly different (Figure 1D). These results confirmed that mitochondrial clustering in basal and parabasal keratinocytes of lesional skin was altered compared with nonlesional skin. This was a result of changes to mitochondrial organization in vitiligo skin and was not a consequence of differences in melanin content.

### scRNA-Seq reveals unique keratinocyte cell states enriched in vitiligo lesional skin.

**Figure 2:**
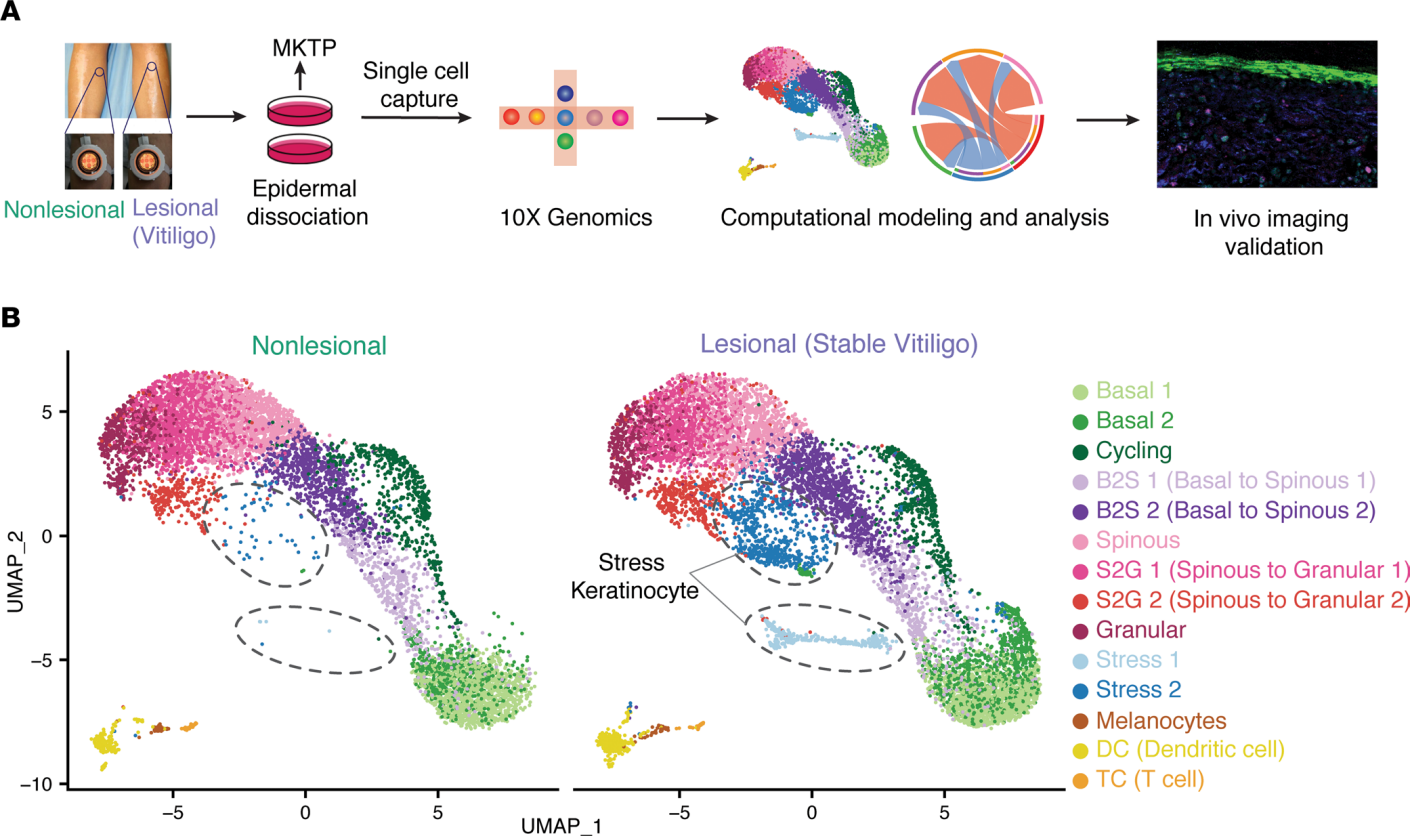
Single-cell isolation of nonlesional and lesional skin of vitiligo patients for scRNA-Seq. (**A**) Schematic diagram of single-cell isolation and scRNA-Seq data analyses. (**B**) UMAP plot of the cells from all patients in both nonlesional (left) and lesional skin (right).

**Figure 3:**
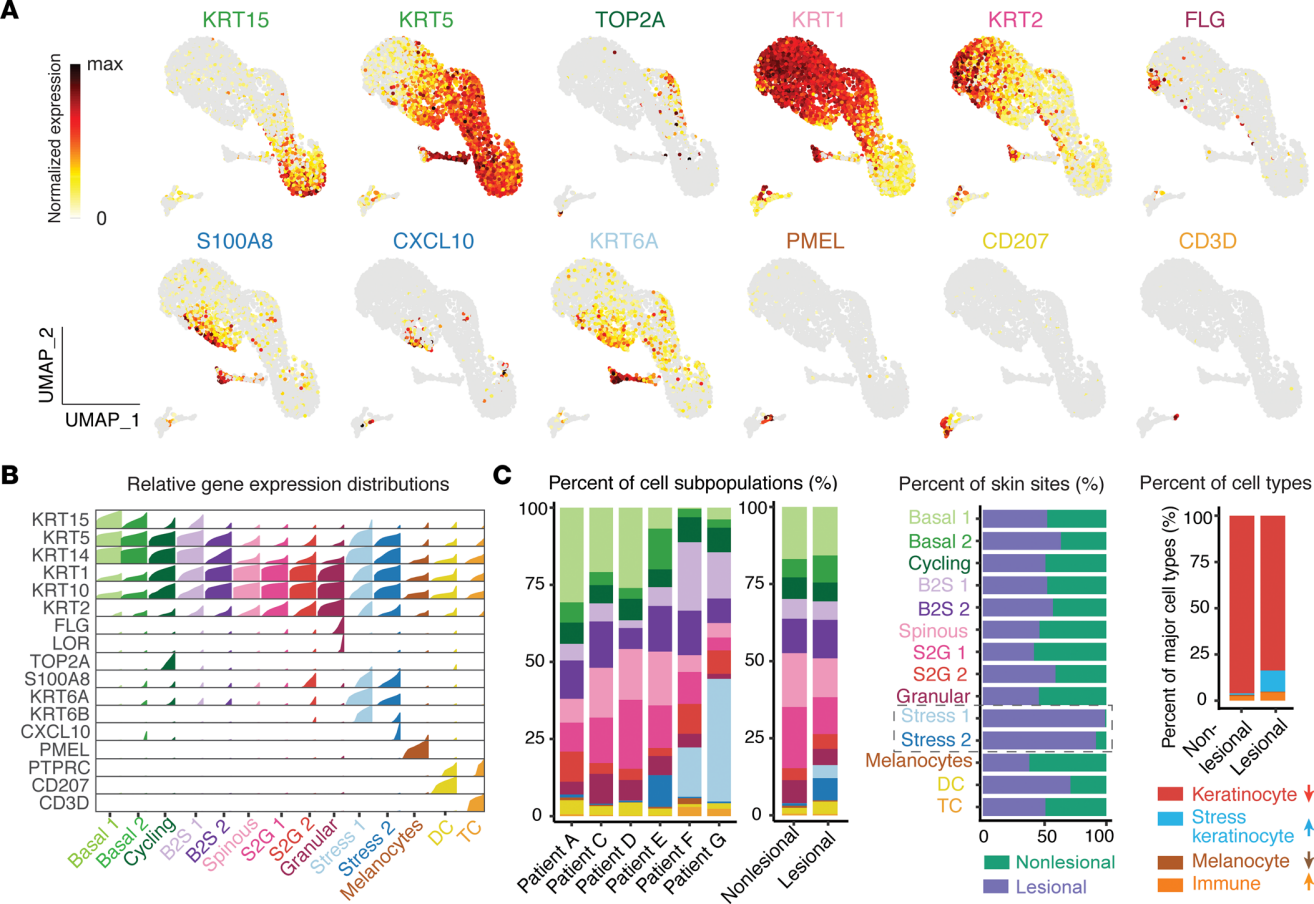
scRNA-Seq analyses of lesional and nonlesional skin reveal unique keratinocyte cell populations in vitiligo patients. (**A**) Feature plots showing expression of the selected markers in the UMAP space of all cells. (**B**) High-density plot showing relative gene expression of key marker genes in different cell subpopulations. Each density plot is composed by bar charts, and bar height corresponds to the relative expression level of the gene in cells that is ordered from low to high. (**C**) Percentages of cell subpopulations across patients, lesional skin, and nonlesional skin (left). Comparison of the percentages of each cell subpopulation across lesional and nonlesional skin (middle). Comparison of the percentages of major cell types including keratinocytes, stress keratinocytes, melanocytes, and immune cells across lesional and nonlesional skin (right). The bar plot shows that the percentages of keratinocytes and melanocytes decrease, while the percentages of stress keratinocytes and immune cells increase in lesional skin compared with nonlesional skin.

MPM imaging demonstrated that basal and parabasal keratinocytes in vitiligo lesions were metabolically altered, suggesting that keratinocyte cell states are different in vitiligo patients. To systematically examine the major keratinocyte cell state changes in vitiligo, we performed scRNA-Seq on a separate group of patient-matched lesional and nonlesional suction blisters from 7 patients using the 10x Genomics Chromium platform (Figure 2A). One set of samples (patient B) was excluded from further analyses due to the low viability of cells (Supplemental Table 2). We performed read depth normalization and quality control (Supplemental Figure 2) and obtained a total of 9254 cells of vitiligo lesional skin and 7928 cells of nonlesional skin for downstream analyses. We performed integration analysis of data from all patients using our recently developed approach scMC, which is designed to preserve biological signals while removing batch effects (28). Unsupervised clustering analysis identified 14 cell clusters (Figure 2B). Using the differentially expressed gene signatures, we were able to attribute clusters to their putative identities (Figure 3, A and B), including basal keratinocytes (high KRT15 and KRT5 expression), spinous keratinocytes (high KRT1 expression), granular keratinocytes (high FLG and LOR expression), cycling keratinocytes (high TOP2A expression), melanocytes (high PMEL expression), T cell (high CD3D expression), and DC (high CD207 expression) (Figure 3, A and B). The intermediate keratinocyte states, including basal-to-spinous transition and spinous-to-granular transition, were defined based on the hybrid expression of KRT15, KRT1, and KRT2. Notably, we identified 2 keratinocyte states that upregulate expression of keratins that are not normally expressed in the mature interfollicular epidermis and are associated with insults like wounding and UV injury (Figure 3A) (29, 30). Stress 1 subpopulation was highly enriched for KRT6A, while Stress 2 subpopulation expressed KRT6A at lower levels. Both populations also expressed KRT16 and S100A8/9, alarmins associated with local inflammation that have been used as biomarkers for other inflammatory conditions (31). We term these populations stress keratinocytes, as their transcriptional signature corresponds with injuries and inflammation. Interestingly, stress keratinocytes were only enriched in vitiligo lesional skin (Figure 2B). Detailed analysis of the 2 immune cell subpopulations T cells and DC showed that they were distinguished from each other with clearly distinct gene signatures and biological processes (Supplemental Figure 3). Cellular composition analysis showed that, although different patients exhibited certain heterogeneity, cell clusters were common among patients (Figure 3C). Compared with nonlesional skin, vitiligo lesional skin showed a dramatically increased presence of stress keratinocyte and, to a lesser extent, of DC, as well as a clear decrease of melanocytes (Figure 3C). Overall, the percentages of keratinocytes and melanocytes were decreased, and stress keratinocytes and immune cells were increased in vitiligo lesional skin (Figure 3C). Moreover, we analyzed keratinocytes from normal human skin using a previously published scRNA-Seq data set where healthy skin was isolated from 4 patients who were undergoing mammoplasty for hypermastia and from 1 patient who had a mastectomy (Supplemental Figure 4A) (32). We did not observe the expression of stress signature genes, suggesting that stress keratinocytes were uniquely enriched in vitiligo lesional skin. To ensure that these differences were not due to different isolation techniques for skin cell isolation, we also analyzed healthy skin generated from suction blisters and found that, similarly, stress keratinocytes were only found in vitiligo skin (Supplemental Figure 8A). Integration analysis using a Seurat package produced similar cellular compositions but did not preserve biological variation, as well. In particular, stress keratinocytes were intermixed with other keratinocyte cell states and were in a spread distribution in the uniform manifold approximation and projection (UMAP) space (Supplemental Figure 5). Collectively, these data provide the first general overview to our knowledge of the major changes in cellular compositions from nonlesional skin to stable vitiligo lesional skin.

### Stress keratinocytes exhibit altered metabolism with dominant upregulation of OxPhos.

**Figure 4:**
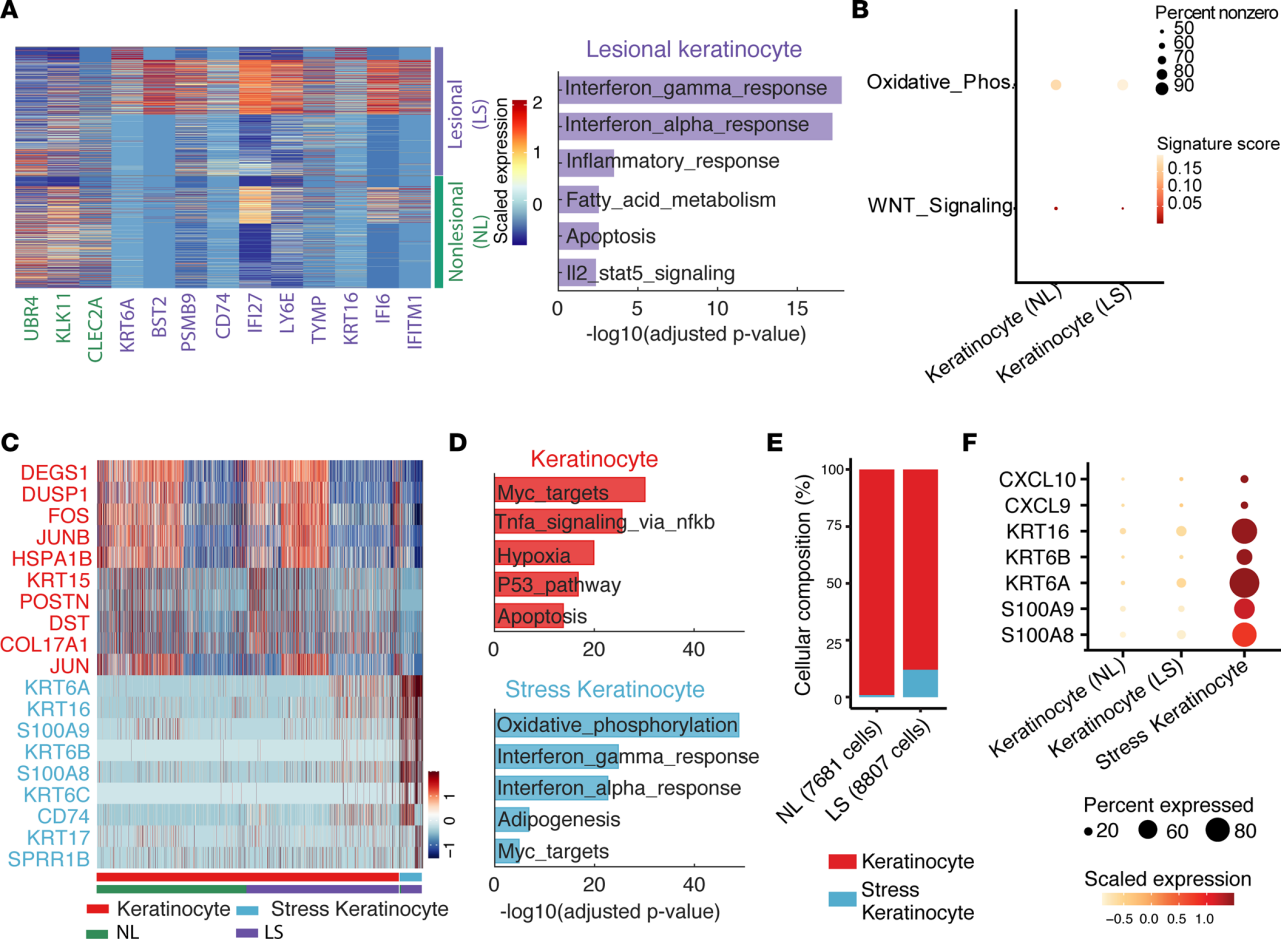
Stress keratinocytes have a unique gene signature and are the main source of CXCL9 and CXCL10. (**A**) Heatmap of scaled expression levels of top 10 differentially expressed genes between nonlesional and lesional keratinocytes and enriched Hallmark pathways of the highly expressed genes in lesional keratinocytes. (**B**) Dot plots of signature scores of WNT signaling and OxPhos pathway between nonlesional and lesional skin. The size represents the percentage of expressing cells, and colors indicate the scaled signature scores. (**C**) Heatmap of scaled expression levels of differentially expressed genes between stress keratinocytes and other keratinocytes. (**D**) Enriched Hallmark pathways of highly expressed genes in stress keratinocytes and other keratinocytes, respectively. (**E**) The composition of stress keratinocytes and other keratinocytes in nonlesional and lesional skin. (**F**) Dot plot of stress associated markers in nonlesional skin, lesional skin, and stress keratinocytes. The size represents the percentage of expressing cells, and colors indicate the scaled expression.

To further characterize keratinocyte differences in detail between vitiligo lesional and nonlesional skin, we first performed differential expression analysis and found that lesional skin expressed higher levels of *KRT6A* and *KRT16* keratins that are not normally expressed in the mature interfollicular epidermis and are associated with insults like wounding and UV injury (Figure 4A) (29, 30). Inflammatory and immune response–related genes such as CD74, IFI27, IFI6, and IFITM1 were also significantly increased, and this was further confirmed by the hallmark pathway enrichment analysis of the genes highly expressed in vitiligo lesional skin using the Molecular Signatures Database (MSigDB; Figure 4A) (33). In addition, we found that the top 2 enriched pathways were IFN-γ and IFN-α responses (Figure 4A), and this is consistent with previous findings that lesional keratinocytes differed from their nonlesional counterparts in upregulation of IFN responses (Figure 4A) (5, 34). Gene scoring analysis revealed downregulation of WNT signaling (Figure 4B), consistent with the known role of WNT in melanocyte pigmentation (5, 34). Since MPM demonstrated metabolic differences between nonlesional and lesional vitiligo skin, we further computed the signature scores of OxPhos. Interestingly, higher scores were observed in lesional skin (Figure 4B).

**Figure 5:**
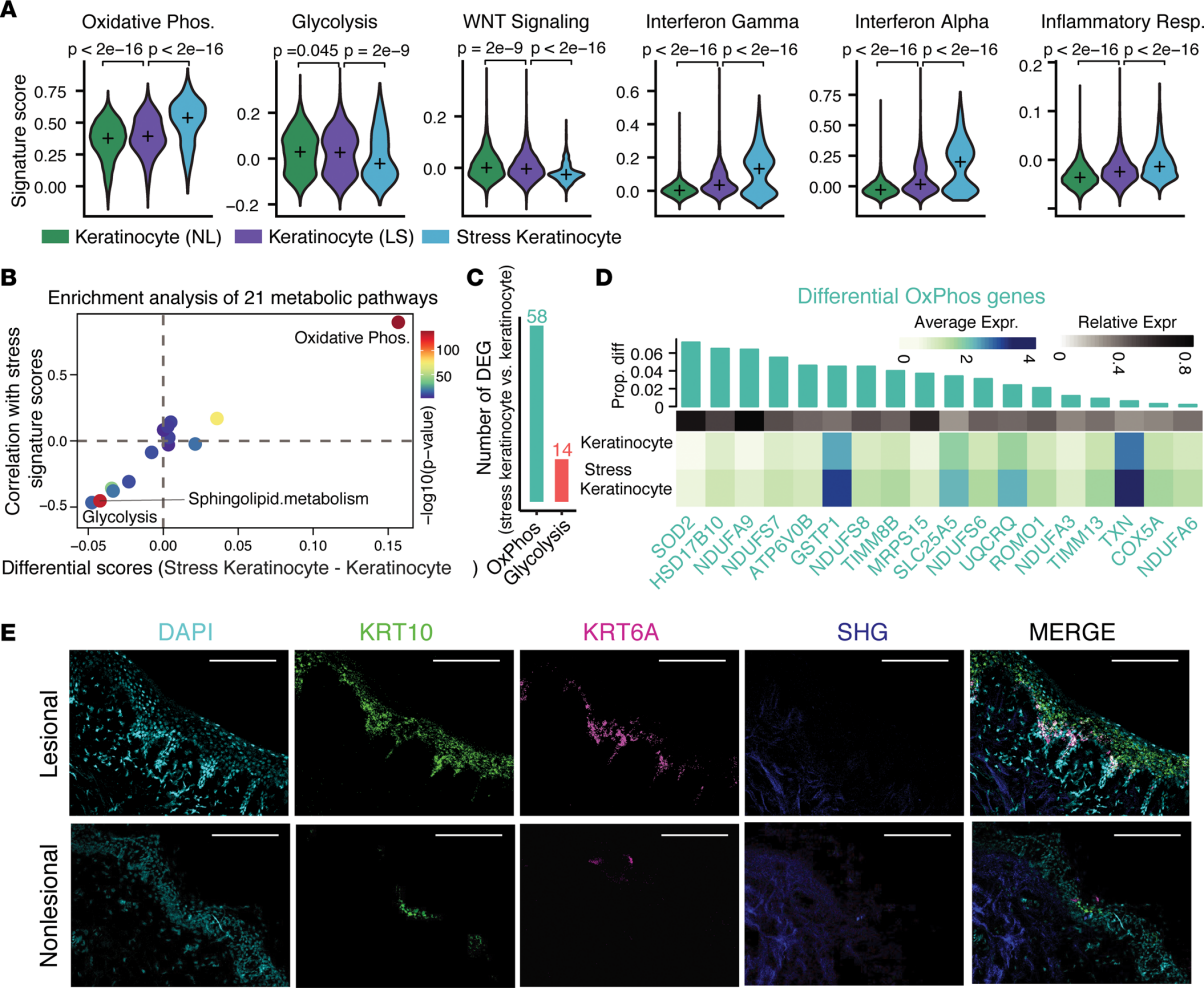
Stress keratinocytes have altered energy utilization and shift toward oxidative phosphorylation. (**A**) Violin plots of signature scores of OxPhos, glycolysis, WNT signaling, IFN-γ, IFN-α, and inflammatory response across nonlesional skin, lesional skin, and stress keratinocytes. The 2-sided Wilcoxon rank-sum test was used to evaluate whether there are significant differences in the computed signature scores. (**B**) Enrichment analysis of 21 metabolic pathways in stress keratinocytes versus other keratinocytes. Each dot represents 1 pathway. The *x* axis represents the differential gene signature scores of each metabolic pathway between stress keratinocytes and other keratinocytes. The *y* axis represents the Pearson’s correlation of gene signature scores between each metabolic pathway and stress response. Gene signature scores of stress response were computed based on the marker genes of stress keratinocytes. Colors represent the *P* values from 2-sided Wilcoxon rank-sum tests of each gene signature score between stress keratinocyte and other keratinocytes. (**C**) The number of differentially expressed OxPhos- and glycolysis-related genes in stress keratinocytes versus other keratinocytes. (**D**) Heatmap showing the average expression of top 18 differentially expressed OxPhos-related genes between stress keratinocytes and other keratinocytes. The top green bars represent the difference in the proportion of expressed cells between stress keratinocytes and other keratinocytes. (**E**) RNAscope demonstrating Krt6A and Krt10 in situ hybridization in patient-matched lesional and nonlesional punch grafting tissue. DAPI (cyan) was used to stain nuclei and second harmonic generation (blue) demonstrating location of collagen.

To figure out whether the above observed differences in signaling and metabolism were attributed to the unique stress keratinocytes in vitiligo lesional skin, we next focused on the difference between keratinocytes and stress keratinocytes. Differential expression analysis revealed distinct gene signatures between these 2 keratinocyte states (Figure 4C). In addition to *KRT6*, *KRT16*, *KRT17,* *S100A8*, and *S100A9* alarmins are known to be expressed in stress keratinocytes (Figure 4C) (35). Hallmark gene enrichment analysis of the differentially expressed genes showed that stress keratinocytes were enriched by OxPhos and IFN responses (Figure 4D). Since there were nearly no stress keratinocytes in nonlesional skin (Figure 4E), we focused on 3 keratinocyte groups: nonlesional keratinocytes, lesional keratinocytes, and lesional stress keratinocytes. Comparison of these groups showed that CXCL9/10, KRT16, KRT6A/B, and S100A8/9 were specifically expressed in stress keratinocytes instead of other 2 keratinocyte groups (Figure 4F). We further performed quantitative comparison of these 3 keratinocyte groups using gene scoring analysis. Impressively, we observed dramatic differences between stress keratinocytes and both lesional and nonlesional keratinocytes, in terms of OxPhos, glycolysis, WNT signaling, IFN-γ, IFN-α, and inflammatory response (Figure 5A). Notably, significantly increased OxPhos and decreased glycolysis were consistent with our MPM imaging data (Figure 5A and Figure 1A). These results suggest that stress keratinocytes in vitiligo lesional skin dominantly account for the observed differences in signaling and energy utilization between lesional and nonlesional skin.

To further examine whether OxPhos and glycolysis were the prominently impaired metabolic processes in vitiligo lesional skin, we quantitatively evaluated the enrichment of 21 metabolic pathways using gene scoring analysis. We observed that OxPhos and glycolysis were the most significantly altered pathways among all 21 metabolic pathways that showed the largest differences between stress keratinocytes and other keratinocytes and the strongest correlations with stress signatures (Figure 5B). Of note, OxPhos and glycolysis were highly positively and negatively correlated with stress signatures, respectively. There are 58 and 14 differently expressed OxPhos- and glycolysis-related genes, respectively, between stress keratinocytes and other keratinocytes (Figure 5, C and D). Stress keratinocytes were enriched for genes associated with OxPhos, including SOD2, NDUFA9, and ATP6V0B. In contrast, keratinocytes expressed higher levels of genes associated with glycolysis, including ALDH3A2, SDC1, and HSPA5. These results, combined with MPM data, indicate that a subpopulation of cells in vitiligo skin have altered energy utilization and shift toward Oxphos.

We then performed RNAscope on patient-matched lesional and nonlesional skin to localize this keratinocyte population using KRT6A, as it is highly expressed in this population (Figure 5C). We found that, consistent with our MPM imaging, KRT6A-expressing cells were enriched in the basal layer of the epidermis, and more KRT6A-expressing cells were observed in lesional skin (Figure 5E).

### Analysis of cell-to-cell communication reveal major signaling changes in response to vitiligo.

**Figure 6:**
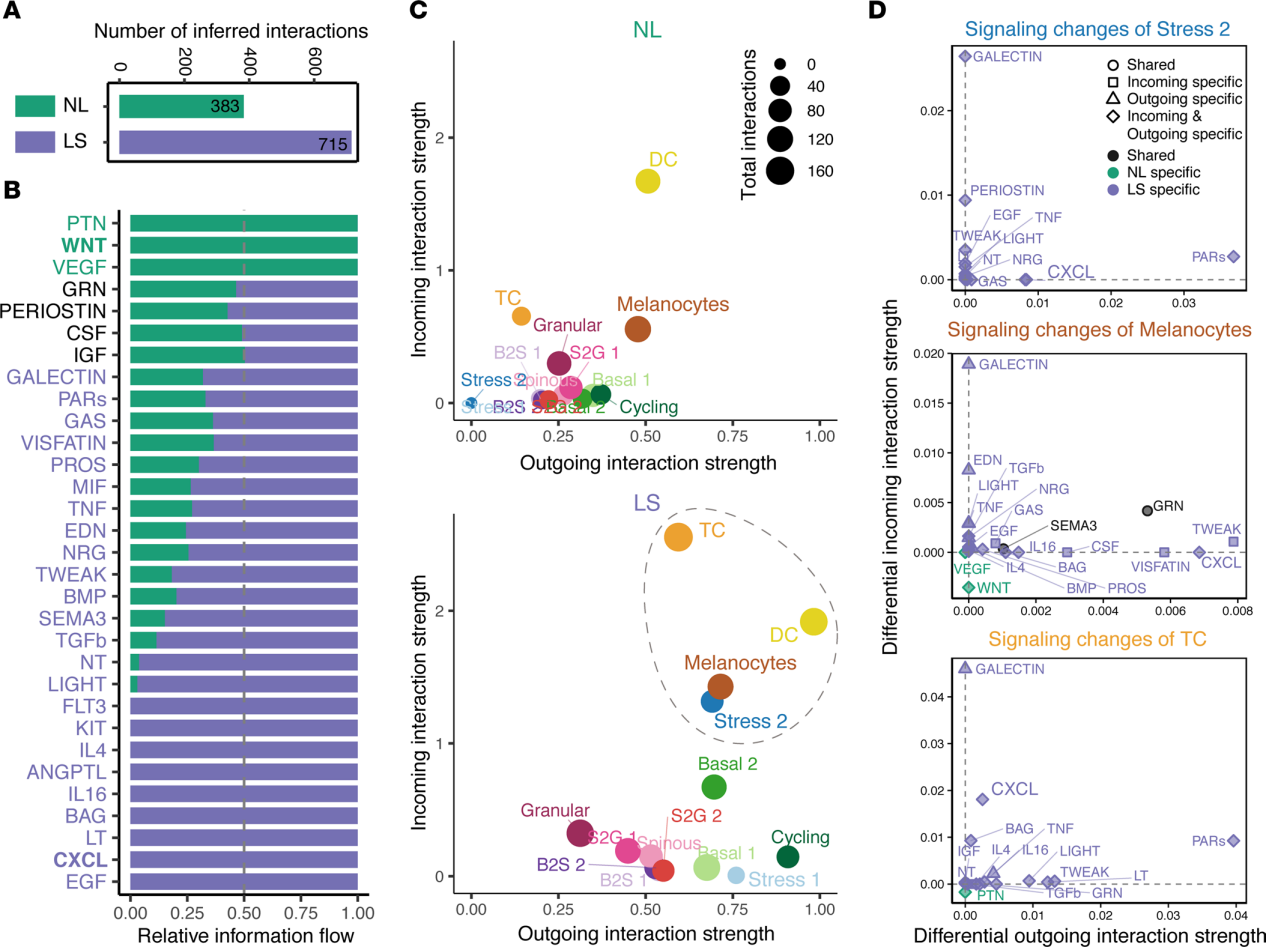
Cell-to-cell communication analysis reveals major signaling changes between nonlesional and lesional vitiligo skin. (**A**) Number of inferred interactions among all cell subpopulations between nonlesional (NL) and lesional (LS) skin. (**B**) The relative information flow of all significant signaling pathways within the inferred networks between nonlesional and lesional skin. Signaling pathways labeled in green represent pathways enriched in nonlesional skin, the middle ones colored by black are equally enriched in both nonlesional and lesional skin, and the ones colored by purple are more enriched in lesional skin. (**C**) Visualization of outgoing and incoming interaction strength of each cell subpopulation in the inferred cell-to-cell communication network of nonlesional (top) and lesional skin (bottom). The dot sizes are proportional to the number of total interactions associated with each cell subpopulation. Dashed circle indicates the most altered cell subpopulations when comparing the outgoing and incoming interaction strength between nonlesional and lesional skin. (**D**) The signaling changes associated with the 3 most altered cell subpopulations.

To systematically detect major signaling changes in stable vitiligo lesions, we applied our recently developed tool CellChat (36) to the scRNA-Seq data of both nonlesional and lesional skin. We observed increased cellular interactions in lesional skin compared with nonlesional skin (Figure 6A). To study the prominent signaling pathways that contribute to the increased signaling in lesional skin, we compared each signaling pathway between nonlesional and lesional skin using the concept of information flow defined as a sum of the communication probability among all pairs of cell groups. We found that several pathways were only activated in nonlesional skin (Figure 6B), including WNT, PTN, and VEGF, consistent with the role of WNT activation in regulating melanocyte differentiation (37). In contrast, many inflammatory pathways prominently increase their information flow at lesional skin as compared with nonlesional skin, such as CXCL, IL-4, IL-6, LT, LIGHT, TWEAK, TNF, VISFATIN, and GALECTIN. Intriguingly, we also observed increased KIT signaling in lesional skin, suggesting that loss of this melanocyte homeostatic signal alone is not responsible for the failure of chronic vitiligo lesions to repigment.

To see which cell subpopulations contribute to the altered signaling in lesional skin, we next studied how different cell subpopulations changed their signaling patterns in nonlesional versus lesional skin using network centrality analysis, which computes the outgoing and incoming interaction strength of each subpopulation to represent the likelihood as signaling sources and targets, respectively. This analysis revealed that T cells emerged as major signaling targets while DC became dominant signaling sources. Melanocytes and Stress 2 keratinocytes also prominently increased their outgoing and incoming signaling from nonlesional to lesional skin (Figure 6C), likely accounting for increase intercellular interactions (Figure 6A). We then asked which signaling pathways contributed to the signaling changes of these populations. Differential interaction analysis showed that the prominently increased outgoing signaling of Stress 2 keratinocytes and melanocytes and the incoming signaling to T cells was CXCL (Figure 6D), suggesting that the CXCL signaling pathway was the dominantly dysfunctional signaling sent from Stress 2 keratinocytes and melanocytes to T cells. Of note, WNT is the major decreased incoming signaling of melanocytes.

**Figure 7:**
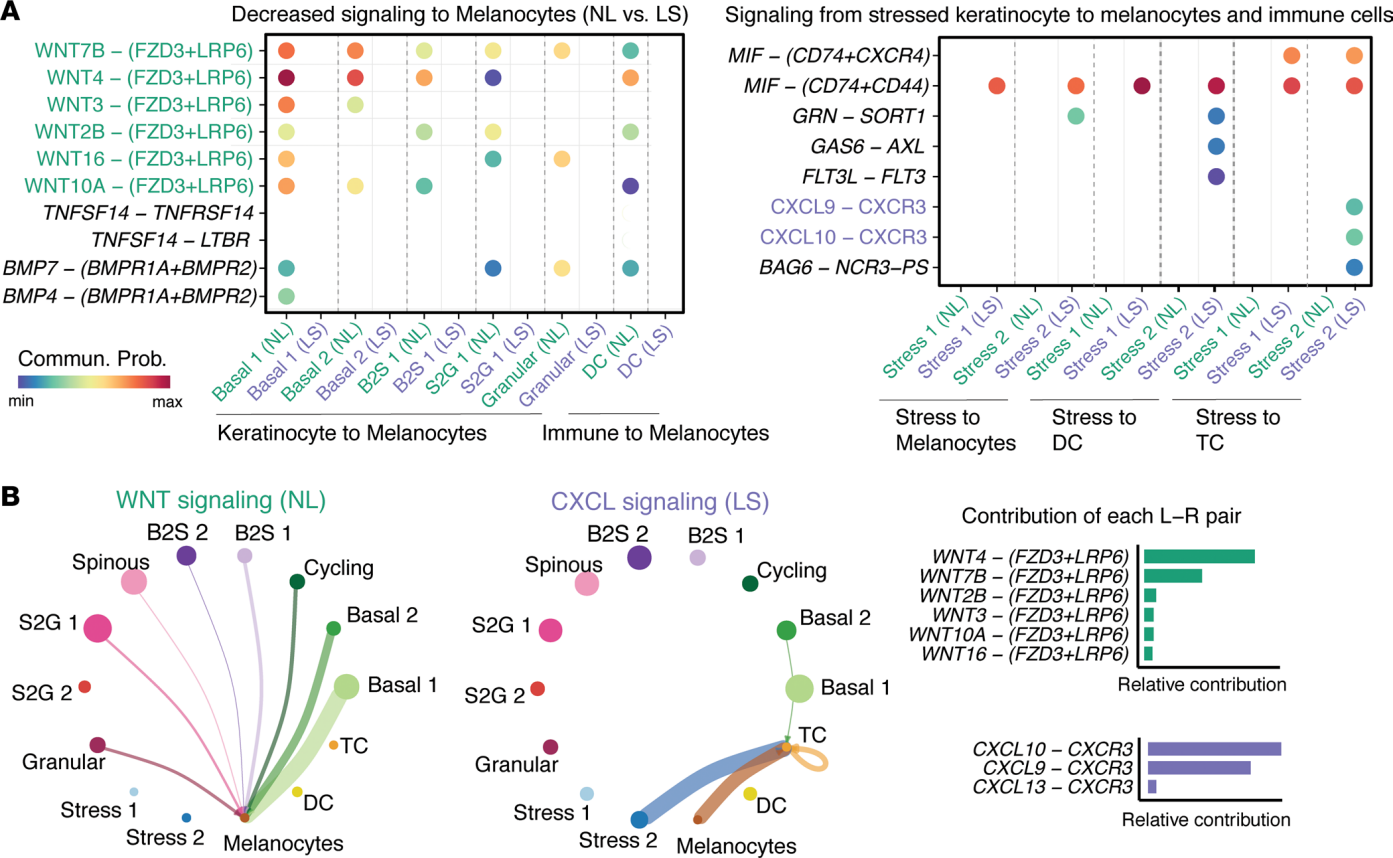
Keratinocyte-melanocyte and keratinocyte–immune cell communication is altered in lesional vitiligo skin compared with nonlesional skin. (**A**) Bubble plot in left panel shows the decreased signaling from keratinocyte and immune subpopulations to melanocytes (nonlesional versus lesional skin). Bubble plot in right panel shows all significant signaling from stress keratinocyte to melanocytes and immune subpopulations. (**B**) Inferred cell-to-cell communication networks of WNT and CXCL signaling in nonlesional and lesional skin, respectively. The edge width is proportional to the inferred communication probabilities. The relative contribution of each ligand-receptor pair to the overall signaling pathways.

By studying the signals sent to melanocytes, we found that a relative deficiency of WNT and BMP signaling was noted in keratinocytes and DC in lesional skin. In particular, WNT signal was seen in all keratinocyte populations in nonlesional skin with WNT4 and WNT7B driving the signaling (Figure 7, A and B). For the signaling from stress keratinocyte to melanocytes, DC, and T cells, macrophage migration inhibitory factor (MIF) and CXCL signaling were highly active in lesional skin. Notably, for the signaling from stress keratinocyte to T cells, ligands CXCL9 and CXCL10 and their receptor CXCR3 were found to be uniquely active in lesional skin (Figure 7, A and B). Interestingly, while increased MIF signaling was seen in both Stress 1 and Stress 2 keratinocytes, the increase in CXCL signaling was only seen in Stress 2 keratinocytes. Taken together, our analyses indicated the prominent alteration of cell-to-cell communication networks in vitiligo lesional skin and predicted major signaling changes that might drive vitiligo pathogenesis.

### Pseudotemporal dynamics reveal transition dynamics of stress keratinocytes.

**Figure 8:**
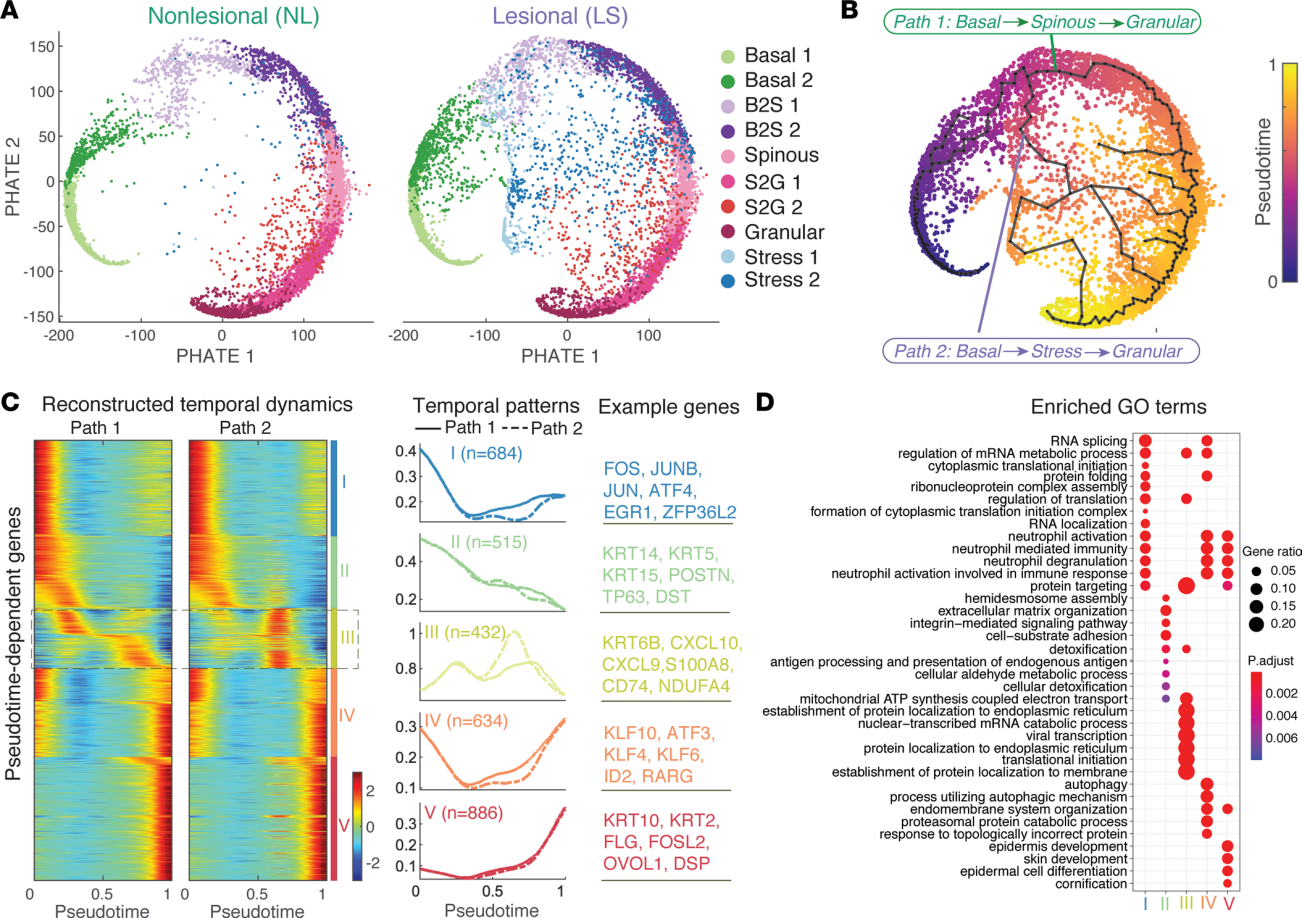
Pseudotemporal dynamics reveal transition dynamics of stress keratinocytes. (**A**) Projection of keratinocytes onto the PHATE space revealed the potential lineage relationships between different keratinocyte subpopulations in nonlesional (NL, left panel) and lesional (LS, right panel) skin. Cells were colored by the annotated cell identity. (**B**) The inferred pseudotemporal trajectories of all cells using Monocle 3. Cells were colored by the inferred pseudotime. Pseudotemporal trajectory analysis revealed 2 potential transitional paths, as indicated by Path 1 and Path 2. (**C**) Pseudotemporal dynamics of all pseudotime-dependent genes along the Path 1 and Path 2. Each row (i.e., gene) is normalized to its peak value along the pseudotime. These genes were clustered into 5 groups with the average expression patterns (middle) and representative genes (right). Solid and dashed lines indicate the average expression of a particular gene group in Path 1 and Path 2, respectively. The number of genes in each gene group is indicated in parenthesis. (**D**) Enriched biological processes of the 5 gene groups in **C**.

To explore the role of stress keratinocytes in keratinocyte differentiation, we performed pseudotemporal trajectory analysis using all keratinocyte cells except for cycling cells from all samples. By applying the diffusion-based manifold learning method PHATE (38, 39) to the batch-corrected data obtained from scMC (28), we observed a differentiation path in the nonlesional skin, recapitulating sequential stages of the keratinocyte differentiation process from basal state to the terminally differentiated granular state. However, in vitiligo lesional skin, in additional to the known keratinocyte differentiation path (Path 1), another potential differentiation path (Path 2) was found to attribute to stress keratinocytes (Figure 8, A and B). Using an unsupervised pseudotemporal trajectory inference tool Monocle 3 (40), we showed that the stress keratinocytes indeed contributed to alternative differentiation paths, indicating a transition from an early intermediate keratinocyte state (basal to spinous transition) to stress keratinocytes, to a late intermediate keratinocyte state (spinous to granular transition), and then to granular state (Figure 8B). Such observation was further confirmed using another trajectory inference approach PAGA (41), showing strong likelihood of the transition between stress keratinocytes and the late keratinocyte states (Supplemental Figure 6A). To further analyze the keratinocyte differentiation dynamics, we performed RNA velocity analysis using scVelo, a computational tool that can predict potential directionality and speed of cell state transitions based on levels of spliced and unspliced mRNA (42). RNA velocity analysis also provided evidence for enhanced transition dynamics from stress keratinocytes to the late keratinocyte state (Supplemental Figure 6B). Together, in addition to the normal keratinocyte differentiation trajectory, these analyses showed that the transition dynamics of stress keratinocytes contribute to an altered keratinocyte differentiation trajectory in vitiligo lesional skin (Supplemental Figure 6C).

**Figure 9:**
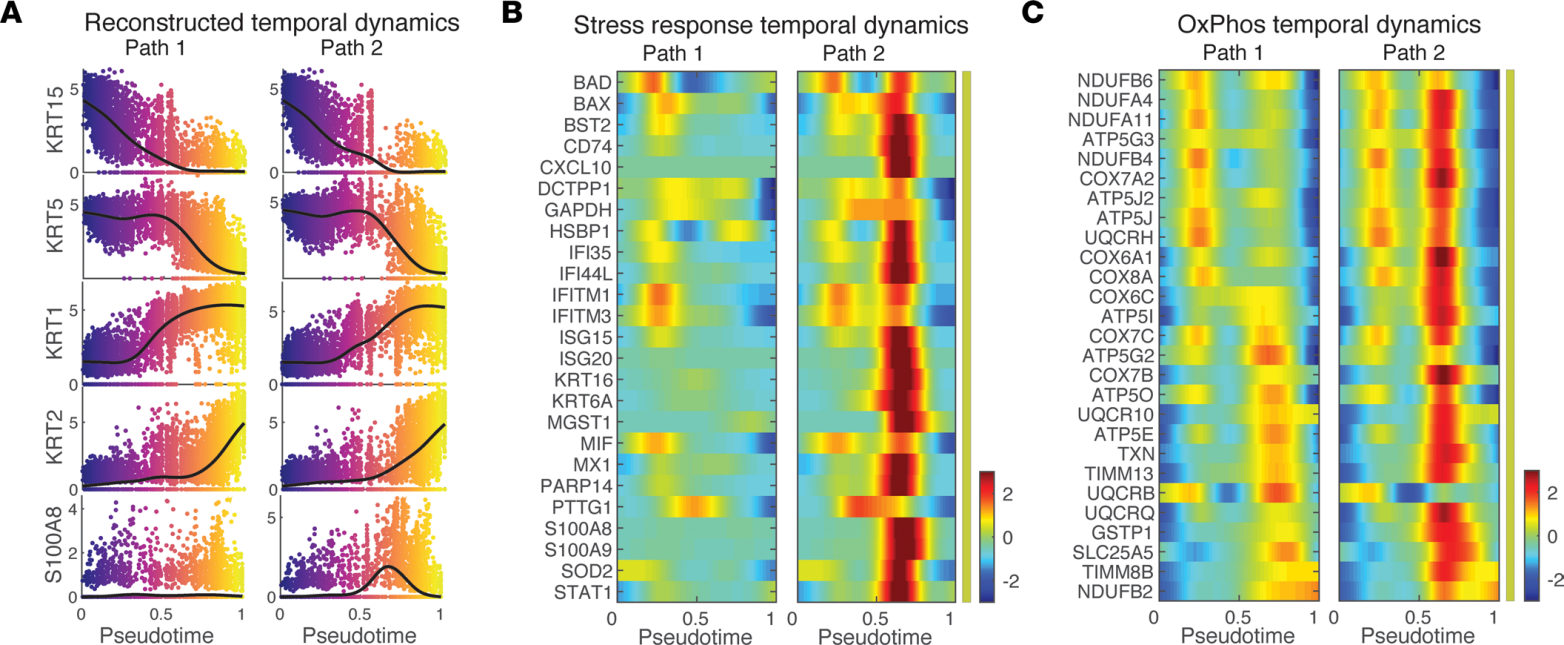
Upregulation of stress response and OxPhos are seen in the reconstructed pseudotemporal dynamics of stress keratinocytes. (**A**) The reconstructed pseudotemporal dynamics of selected marker genes along the inferred pseudotime in Path 1 and Path 2, respectively. Black lines represent the average temporal patterns that were obtained by fitting a cubic spline. Cells were colored by the inferred pseudotime. (**B**) Pseudotemporal dynamics of the pseudotime-dependent genes related with the stress response and along the inferred pseudotime in Path 1 and Path 2. (**C**) Pseudotemporal dynamics of the pseudotime-dependent genes related with OxPhos along the inferred pseudotime in Path 1 and Path 2.

We next sought to identify key molecular changes that may be important for keratinocyte cell state transitions using single-cell energy path (scEpath) (39). scEpath identified 1284 and 3151 pseudotime-dependent genes over the normal (Path 1) and alternative keratinocyte differentiation trajectories (Path 2), respectively (Figure 8C). These pseudotime-dependent genes were further classified into 5 groups based on their pseudotemporal dynamics. Interestingly, the gene group III exhibited distinct expression dynamics along the Path 1 versus Path 2 while the remaining gene groups followed very similar dynamical trends on both trajectories. Genes in group III included not only stress keratinocyte–related signatures such as KRT6B, CXL10, CXCL9, S100A8, and CD74, but also OxPhos-associated signatures such as NDUFA4 and ATP5G3 (Figure 8C). Further gene ontology (GO) enrichment analysis revealed distinct enriched biological processes among these 5 gene groups, including the enriched metabolic processes in group III (Figure 8D). The reconstructed pseudotemporal dynamics of typical maker genes well recapitulated the expected keratinocyte differentiation dynamics (Figure 9A). As expected, we observed stronger activation of stress response, inflammatory response, and OxPhos-associated genes in the Path 2 compared with Path 1 (Figure 9, B and C). Notably, we did not observe changes in expression of transcripts of genes known to be involved in the process of mitochondrial fusion and fission itself (MFN2, OPA1, and DRP1) (data not shown), suggesting that the observed changes were not a result of changes in fission or fusion processes but, instead, a result in changes in NADH metabolism. Taken together, stress keratinocytes induce an altered keratinocyte differentiation trajectory with strong activation of inflammatory response and OxPhos-related gene expression in vitiligo lesional skin.

### MPM imaging of patients undergoing punch grafting demonstrates that keratinocyte metabolic alterations normalize in clinical responders.

**Figure 10:**
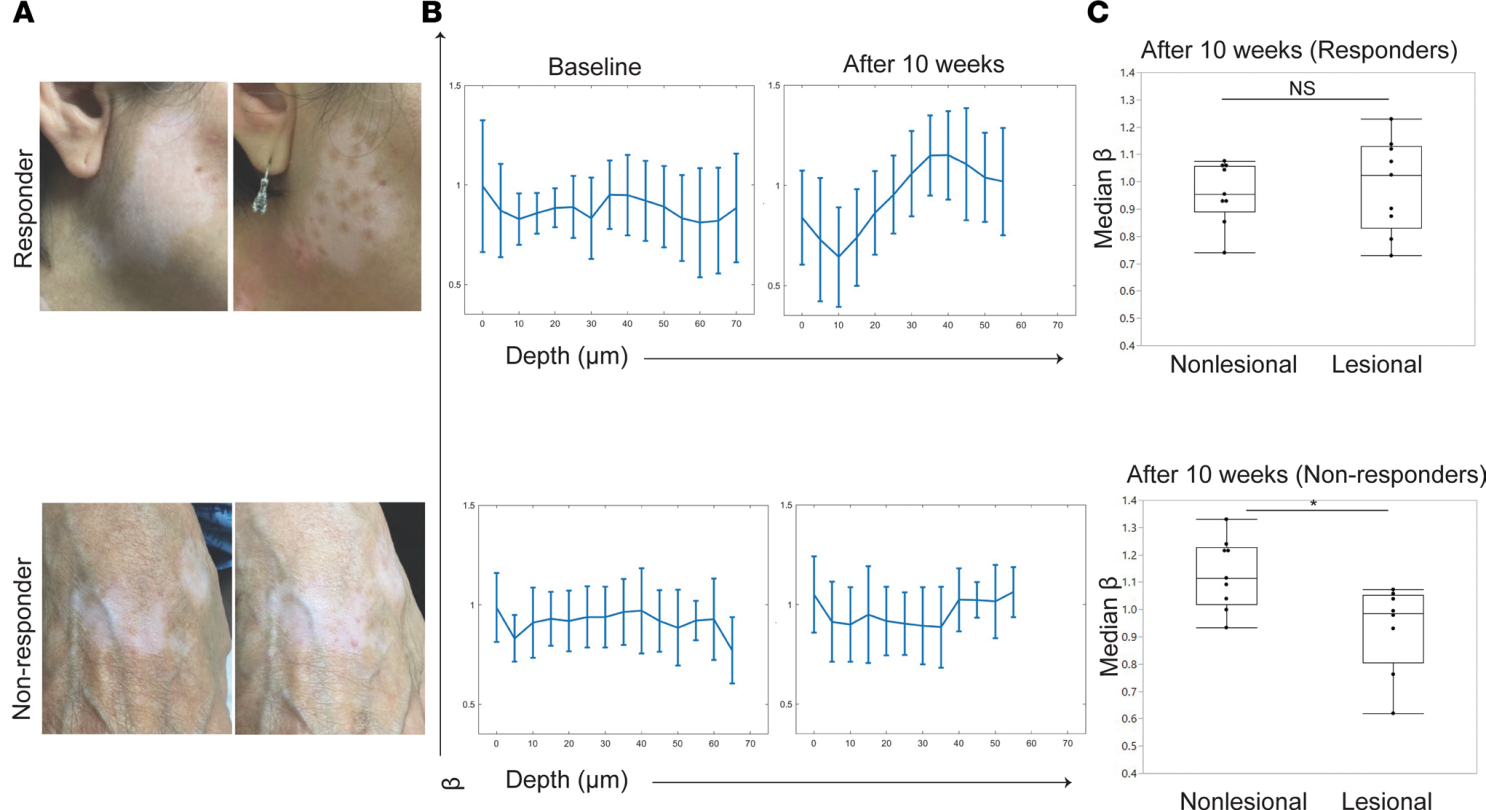
Keratinocyte energy utilization normalize in vitiligo patients who respond to punch grafting treatment but persist in nonresponders. (**A**) Representative clinical images from vitiligo patients undergoing punch grafting treatment. Clinical responder on top and nonresponder on the bottom. (**B**) Average mitochondrial clustering values (β values) based on *Z* stacks from 6 vitiligo patients as a function of depth for responders and nonresponders at baseline are shown as spline fits. Patients were followed and imaged again after 10 weeks. Average mitochondrial clustering values (β values) for clinical responders (*n* = 3) and nonresponders (*n* = 3) are shown. (**C**) Distribution of β variability values (right) in punch grafting responders and nonresponders (*n* = 6); each value corresponds to a *Z* stack of images acquired. *P < 0.05 by *t* test.

Our noninvasive imaging data and scRNA-Seq suggest that it is feasible to use MPM to track keratinocyte populations favoring OxPhos in patients with vitiligo. We followed stable vitiligo patients undergoing a combination of punch grafting, a procedure where autologous small punch grafts are harvested from nonlesional skin and deposited into lesional skin, and phototherapy treatment to determine how stress keratinocytes change by imaging skin immediately adjacent to the graft site with MPM at baseline and 10 weeks after treatment. In patients who responded to treatment and demonstrated repigmentation (Figure 10A, top), keratinocyte mitochondrial clustering values (β) in graft perilesional skin resembled nonlesional skin after treatment (Figure 10B), and the epidermal depth–dependent shift toward glycolysis at the basal layer was restored (Figure 10C). In contrast, clinical nonresponders (Figure 10A) had persistent changes in mitochondrial clustering values in graft perilesional skin (Figure 10B) similar to vitiligo lesional skin at baseline (Figure 1A). The epidermal depth–dependent shift toward OxPhos seen in baseline vitiligo lesional skin remained stable (Figure 10C), suggesting that metabolically altered stress keratinocytes persisted in clinical nonresponders. These findings suggest that the presence of stress keratinocytes with shifted metabolism is associated with a lack of clinical response.

### Stress keratinocytes are not seen in acute vitiligo skin.

Our scRNA-Seq data suggest that stress keratinocytes play a role in stable vitiligo disease persistence, but whether similar populations exist in active vitiligo is unknown. To see if similar keratinocyte populations exist in active disease, we analyzed a recently published data set from 10 active vitiligo patients who used a similar suction blister approach to isolate lesional and nonlesional samples (43). The published data set also had 7 healthy skin samples generated by suction blisters for comparison. We used the original annotated cell types (Supplemental Figure 7A) in the published data set and looked at the express of stress keratins (*KRT6A*, *KRT16*), *S100A8/9*, and *CXCL9/10* (Supplemental Figure 7, B and C) and found that a small subset of cells in the KRT-ECR cluster expressed stress keratins but not other markers of stress keratinocytes. The KRT-ECR cluster from the active vitiligo data set consisted of 357 cells, but the majority were from nonlesional vitiligo skin (245 cells) and healthy skin (108 cells). Active vitiligo lesional skin only contributed 4 cells to the KRT-ECR cluster. This observation contrasts our data where lesional vitiligo skin accounted for most of the stress keratinocytes (Figure 2B and Figure 3C).

To further explore the differences between active and stable vitiligo cellular populations, we integrated the 2 data sets using the original annotated cell types. Consistent with our analysis of healthy skin from a separate data set (Supplemental Figure 4), stress keratinocytes were not observed in healthy skin samples generated from suction blisters (Supplemental Figure 8A). Again, *KRT6A-* and *KRT16-*expressing cells were found in healthy and nonlesional acute vitiligo skin, but these populations did not express *CXCL9/10* (Supplemental Figure 8B). We also computed similarity scores between the cell types in active and stable vitiligo data sets and found that they shared other keratinocyte, melanocyte, and immune populations (Supplemental Figure 8C). However, Stress 1 and Stress 2 populations were unique in stable vitiligo and expressed the highest levels of *CXCL9/10* (Supplemental Figure 8D). We also looked at the metabolic score in the different cell populations using the same approach (Figure 5A) and did not find the same metabolic alterations seen in stable vitiligo stress keratinocytes (Supplemental Figure 9).

## Discussion

To date, the study of human vitiligo and cell-to-cell interactions in the tissue microenvironment (TME) have largely been limited to traditional in vitro cultures and IHC methods due to the lack of tools to assess cellular changes in situ. Here, we combine MPM in vivo imaging of stable vitiligo patients and various scRNA-Seq analyses to demonstrate that a small subpopulation of stress keratinocytes in the basal/parabasal layer exhibit a unique signature — energy utilization preferences for OxPhos, expression of stress keratins, alarmins, and CXCL9/10 and diminished WNT signaling — and could drive the persistence of white patches in vitiligo. Our data suggest that it is feasible to use MPM as a noninvasive method to track OxPhos-shifted keratinocyte populations in vitiligo. The role of stress keratinocytes in stable vitiligo is further suggested by their persistence in patients who do not respond to punch grafting treatment. Previous studies on metabolic alterations in vitiligo largely focused on melanocytes’ increased susceptibility to oxidative insults such as H_2_O_2_ due to decreased expression of antioxidant pathways (44–46). Oxidative stress led to HMGB1 release by cultured melanocytes, which then stimulates cytokine release by keratinocytes (47). Studies on cultured keratinocytes from vitiligo skin showed swollen mitochondria and similar increased susceptibility to oxidative stress (11, 48). However, definitive studies looking at keratinocyte energy utilization and its contributions to vitiligo have been lacking. Our study addresses this gap by first using MPM to identify keratinocyte mitochondrial changes in stable vitiligo patients and then corroborating these findings with scRNA-Seq to demonstrate that specific basal and parabasal keratinocyte states exhibit increased OxPhos and communicate with T cells via the CXCL9/ CXCL10/CXCR3 axis and exhibit decreased WNT signaling to melanocytes.

Most studies on vitiligo have focused on active disease, and the importance of the CXCL9/CXCL10/CXCR3 axis is well established from studies on human skin samples (4, 8, 34, 49). Stable vitiligo, however, remains enigmatic (50). Transcription analyses on depigmented whole skin show minimal immune activation with no *CXCL10* elevation (5). Flow cytometry of stable vitiligo skin blisters demonstrated the presence of a small population of melanocyte-specific CD8^+^ resident memory T cells (T_RM_), and depletion of T_RM_ by targeting CD122 led to repigmentation in a mouse model of vitiligo (51). By using scRNA-Seq to identify changes in cellular compositions in stable vitiligo skin, we identified a keratinocyte state with transcriptome changes important in communicating with other cell types to drive disease persistence. The signals from stress keratinocytes were likely lost from averaging cell gene expression in previous whole skin transcriptional studies, accounting for observed differences in CXCL10 expression in our study (5, 52, 53). By utilizing CellChat analyses, our data highlight that, in stable vitiligo, a small epidermal niche of metabolically altered stress keratinocytes communicate with T cells and melanocytes to form local inflammatory circuits to drive disease persistence (Figure 6), highlighting that vitiligo likely involves multiple etiologic factors (54). We also compared our data to a recently published study looking at active vitiligo and found that the Stress 2 keratinocytes expressing CXCL10 were unique to stable vitiligo (43). In the active vitiligo data set, keratinocytes, DCs, and macrophages were strong producers of CXCL10, while in stable vitiligo, stress keratinocytes were the only source of CXCL10. This suggests that the contexts in which CXCL9/10 are produced in active and stable vitiligo are different. Whether this is due to the presence of more activated T cells in active disease, leading to increased immune CXCL contribution, will need to be further evaluated. While some keratinocytes in the active vitiligo data set expressed stress keratins (*KRT6A*, *KRT16*), they were mainly derived from healthy and nonlesional skin. Metabolic alterations were not apparent in the active vitiligo data set. Keratinocytes as drivers of local inflammatory loops have been suggested in atopic dermatitis and psoriasis (40). We show that similar loops are important in vitiligo persistence and that a population of keratinocytes derived from basal keratinocytes secrete chemokines to communicate with T cells and lack Wnt signals to inhibit melanocyte migration and repopulation. How stress keratinocytes are established in the first place and whether they play a key role in the maintenance of this cellular circuitry remain obscure. Are stress keratinocytes a consequence of intrinsic keratinocyte differentiation defects in genetically susceptible individuals? Or do extrinsic signals from other tissue cells (T cells, the absence of melanocytes, or a combination) drive this cellular state? Studies are currently underway to investigate when metabolically altered keratinocytes first appear and how they may affect the repigmentation process in patients undergoing treatment.

The findings of our study raise the possibility of targeting keratinocyte energy utilization in vitiligo treatment. Intriguingly, biguanides such as phenformin and metformin that inhibit OxPhos have been shown to affect keratinocyte differentiation and pigmentation (55, 56). Whether these drugs will also inhibit keratinocyte-derived signals that affect immune cell and melanocyte recruitment is unclear and represents an unexplored area for drug targeting in vitiligo. Interestingly, stress keratinocytes expressing *KRT6*, *KRT16*, and *S100A8/9* have been identified in the human epidermis of psoriasis and melanomas, raising the possibility that they can play a wide variety of roles in the diseased skin TME (57–59). Further studies on stress keratinocytes will improve our understanding of how keratinocyte states affect the TME and contribute to disease pathogenesis.

A caveat to our study is that scRNA-Seq analyses were performed on skin blisters that do not include fibroblasts and other dermal cell types. We chose blisters, as they represent a nonscarring method to collect vitiligo skin samples and had previously been shown to be sufficient to predict disease activity (4). The absence of dermal tissue in our analysis may account for the lack of innate immune cells that other groups have identified (60, 61). However, a recent study comparing scRNA-Seq analyses of cells from suction blister and punch biopsy found that the 2 methods were comparable in pathway analysis (62). Suction blistering allowed for improved resolution of epidermal cell types, although there were some variations in cellular subtypes. Detailed analysis of vitiligo skin has been hampered by the lack of fresh tissue samples for analysis, as induction of blisters or biopsy itself can induce the disease (1). Moreover, patient-to-patient variation in vitiligo can be significant, which makes it difficult to make generalized conclusions on the pathogenesis of the disease. Here, we have coupled together imaging of lesional and nonlesional skin with scRNA-Seq analysis that specifically controls for sample-to-sample and patient-to-patient variability (scMC) to make generalizable conclusions regarding disease pathogenesis, providing a roadmap for the study of other diseases that are controlled by cell-to-cell interactions in tissue.

Our data indicate that stress keratinocytes have altered energy utilization, drive local inflammation in the skin microenvironment, and can be visualized in situ in human patients using noninvasive MPM imaging. These results are significant because they provide evidence for a potential link between stress keratinocytes and vitiligo persistence. They also indicate that MPM imaging can also be used to follow vitiligo patients longitudinally to better understand the role stress keratinocytes in disease pathogenesis and identify areas that could be targeted by new therapies. These new therapies could range from targeted destruction of altered keratinocytes (laser therapies) or pharmacologic modulation of their physiology. As an example, our work implicates the combination of therapies that reverse keratinocyte metabolic defects and JAK inhibitors as a potentially novel treatment for vitiligo. Studying this process will require the generation of new tissue models to study vitiligo pathogenesis that can overcome the limitations of mouse models. Murine epidermis is thinner than human skin, and melanocytes are present in only select epidermal locations; therefore, current models do not fully capture the tripartite interactions between epidermal melanocytes, keratinocytes, and immune cells in human skin. Development of relevant skin tissue models will enable us to address the mechanistic role of stress keratinocytes in vitiligo disease persistence.

## Methods

### Study design.

This study utilized noninvasive MPM and scRNA-Seq to study patient-matched lesional versus nonlesional skin in stable vitiligo and how intercellular communications are affected in depigmented skin. Imaging, suction blister, and punch skin biopsy of patients were performed under IRB-approved protocols at UC Irvine, and samples were deidentified before use in experiments. Vitiligo skin samples were obtained after examination by board-certified dermatologists. Stable vitiligo lesions were characterized by the absence of koebnerization, confetti-like depigmentation, or trichome lesions and those that have not grown in size for at least 1 year (27). Nonlesional sites were selected as normal-appearing, nondepigmented skin on the thigh when examined by Wood’s lamp.

### Patients for imaging.

Twelve vitiligo patients and 5 volunteers with normal skin were imaged in vivo by MPM. All vitiligo patients had stable vitiligo, defined by no change in size for at least 1 year and do not exhibit features of active vitiligo such as koebnerization, confetti-like depigmentation, and trichome (27). Patients were previously unresponsive to treatment attempts (Supplemental Table 1) and had no treatment in the 3 months before imaging for this study. Vitiligo patient ages were 34–74, with an average age of 56. Vitiligo lesion locations included wrist, hand, leg, arm, face, and neck. Nonlesional pigmented skin was selected after a Wood’s lamp exam on separate body sites or at least 12 cm from the closest depigmented macule. Six patients further underwent punch grafting treatment (Supplemental Table 1) and were imaged again 10 weeks after treatment.

### MPM imaging.

We used an MPM-based clinical tomograph (MPTflex, JenLab) for the in vivo imaging of the vitiligo and normal skin. This imaging system consists of a femtosecond laser (Mai Tai Ti:Sapphire oscillator, sub-100 fs, 80 MHz, tunable 690–1020 nm; Spectra-Physics), an articulated arm with near-infrared optics, and a beam scanning module. The imaging head includes 2 photomultiplier tube detectors used for parallel acquisition of TPEF and second harmonic generation (SHG) signals. The excitation wavelength used in this study was 760 nm. The TPEF and SHG signals were detected over the spectral ranges of 410–650 nm and of 385–405 nm, respectively. We used a Zeiss objective (40×, 1.3 numerical aperture, oil immersion) for focusing the laser light into the tissue. The laser power used was 5 mW at the surface and up to 30 mW in the superficial dermis of the skin. We acquired the MPM data as *Z* stacks of en-face images from the stratum corneum to the superficial dermis. The field of view (FOV) for each optical section was 100 × 100 μm^2^, and the step between the optical sections was 5 μm. We imaged the patients’ vitiligo lesional area, and a normally pigmented area on the upper thigh as control. The rationale for selecting the thigh location as a control site for imaging was based on to the fact that the patients we imaged, being unresponsive to prior treatment of vitiligo, were scheduled for micrografting therapy. Patients who underwent punch grafting treatment were imaged at 10 weeks after treatment at the same location. Imaging locations for healthy volunteers with normal skin were the sun-exposed dorsal forearm and the non–sun-exposed volar upper arm to focus on areas with relatively higher pigment amounts (sun-exposed) and relatively lower pigment amounts (non sun-exposed). Due to the limited FOV of each individual scan, we acquired several stacks of images within each site in order to sample a larger area. Thus, a total of 1872 images was acquired for this study, corresponding to an average of 18 images for each imaging site. Images were 512 × 512 pixels and were acquired at approximately 6 seconds per frame. All images were color coded such that green and blue represent the TPEF and SHG signals, respectively. In MPM imaging of skin, the contrast mechanism is based on TPEF signal from NADH, FAD, keratin, melanin, and elastin fibers (63–65) and on SHG signal from collagen (66). These images were used as a basis for the mitochondrial clustering analysis (see Supplemental Methods).

### Suction blister induction and cell isolation for scRNA-Seq.

The donor skin sites were cleaned with ethanol wipes, and 5 suction blisters (1 cm diameter) were created by applying a standard suction blister device. We unroofed the blisters and used half for melanocyte-keratinocyte transplant procedure (67). The rest of the blisters were incubated in trypsin for 15 minutes at 37°C, followed by mechanical separation and centrifugation at 123*g* for 10 minutes at 4°C to pellet cells. Cells were washed with 0.04% UltraPure BSA:PBS buffer, gently resuspended in the same buffer, and filtered through a 70 μm mesh strainer to create a single-cell suspension. Cells were washed, and viability was calculated using trypan blue. scRNA-Seq was performed by the Genomics High Throughput Sequencing facility at UC Irvine, with the 10x Chromium Single Cell 3′ v2 kit (10x Genomics). None of the patients who were imaged overlapped with the cohort of patients who were analyzed by scRNA-Seq. Details of the single cell data analysis are provided in Supplemental Methods.

### Comparison analysis between stable and acute scRNA-Seq data.

A recently published acute vitiligo scRNA-Seq analysis on suction blisters including healthy, nonlesional, and lesional skin was used for comparison (43). We integrated these data with our scRNA-Seq using scMC (28). Details of the comparison code are available in GitHub (https://github.com/zhanglhbioinfor/Codes_for_paper_scRNA-Seq_vitiligo; commit ID: e06b483).

### Patient samples for RNAscope.

Briefly, 2 mm biopsies were performed on lesional and nonlesional skin as part of punch grafting treatment for 3 patients. Control skin was acquired from tumor excision tips without notable pathology from patients without vitiligo. Skin samples were immediately frozen and embedded in OCT. Tissues were stored at –80°C, and cryosections (10 mm thick) of skin were collected on Fisherbrand Superfrost Plus microscope slides. Sections were dried for 60–120 minutes at –20°C then used immediately or within 10 days. In situ hybridization was performed according to the RNAscope Multiplex Fluorescent Reagent Kit v2 (catalog 320293).

Briefly, slides were fixed in cold 4% PFA for 15 minutes and were then dehydrated in 50%, 70%, and 100% ethanol for 5 minutes each at room temperature (RT). H_2_O_2_ was applied for 10 minutes at RT and treated with protease IV for 30 minutes. C2 and C3 probes were diluted in C1 probes at a 1:50 ratio and incubated for 2 hours at 40°C. C1 probes were detected with TSA-fluorescein (Akoya Biosciences), C2 probes with Opal-620, and C3 probes with Opal-690 (Akoya Biosciences). Before mounting, DAPI was added to label the nuclei. Images were acquired using a Leica SP8 FALCON/DIVE (20× objective, 0.75 NA).

### Code availability.

Code for the scRNA-Seq analysis have been deposited at the GitHub repository (https://github.com/amsszlh/Codes_for_paper_scRNA-Seq_vitiligo).

scMC is publicly available as an R package under the GPL-3 license. Source codes, tutorials, and reproducible benchmarking codes have been deposited at the GitHub repository (https://github.com/amsszlh/scMC) and Zenodo repository (https://doi.org/10.5281/zenodo.4138819).

CellChat is publicly available as an R package. Source codes, as well as tutorials, have been deposited at the GitHub repository (https://github.com/sqjin/CellChat). The web-based CellChat Explorer, including Ligand-Receptor Interaction Explorer for exploring the ligand-receptor interaction database and Cell–Cell Communication Atlas Explorer for exploring the intercellular communications in tissues, is available at http://www.cellchat.org/

### Data and materials availability.

Data have been submitted to the GEO data base, accession no. GSE203262 (https://www.ncbi.nlm.nih.gov/geo/query/acc.cgi?acc=GSE203262).

### Statistics.

Statistical comparisons of median β and β variability were conducted using linear mixed effects models in SAS JMP Pro 14. Variables such as patient number and imaging location were modeled as random effects. Whether an area of skin was lesional or nonlesional was modeled as a fixed effect when comparing metrics of mitochondrial clustering among patients. Whether an area of skin was classified as sun-exposed or non–sun-exposed was modeled as a fixed effect when comparing metrics of mitochondrial clustering among healthy volunteers. The significance level for all statistics was set to α = 0.05, and the 2-tailed *t* test was used to compare groups. 

For scRNA-seq gene score calculations, the 2-sided Wilcoxon rank-sum test was used to evaluate whether there are significant differences in the computed signature scores between two groups of cells.

### Study approval.

All human studies were conducted according to an approved IRB protocol of the UC Irvine (no. 2018-4362), with written informed consent obtained from all patients.

## Author contributions

Conceptualization was contributed by MB, QN, AKG, IG, and BJT. Methodology was contributed by JS, GL, LZ, SJ, JLF, CM, CP, FRD, and PM. Investigation was contributed by JS, GL, LZ, SJ, JLF, SJJ, CP, CM, JK, FRD, and PM. Visualization was contributed by JS, GL, LZ, SJ, JLF, and CP. Funding acquisition was contributed by JS, MB, QN, IG, and AKG. Supervision was contributed by IG, MB, QN, AKG, and BJT. Writing of the original draft was contributed by JS, GL, and LZ. Review and editing was contributed by JS, GL, LZ, SJ, JLF, SJJ, CP, IG, QN, MB, and AKG.

## Supplementary Material

Supplemental data

## Version 1 06/02/2022

In-Press Preview

## Version 2 07/08/2022

Electronic publication

## Version 3 07/18/2022

The typographical error was corrected in the article title
